# DFID commentary on the evaluation of the community response to HIV and AIDS

**DOI:** 10.1080/09540121.2012.714458

**Published:** 2013-06-09

**Authors:** Andrea E. Cook, Anna Seymour

**Affiliations:** a DFID, Evaluation Department, Abercrombie House, Glasgow, UK; b DFID, Human Development Department, London, UK

**Keywords:** AIDS, community response, evaluation

## Abstract

The UK Department for International Development (DFID) has funded communities involved in the HIV response for many years. The critical importance of this response, the ownership by and empowerment of communities that has emerged, is to be celebrated. But there is still much we do not know about the complex inter-relationship between community engagement and impact on HIV outcomes. We need a much clearer understanding of how best to support and sustain effective and efficient responses. The DFID funded World Bank evaluation of the community response to HIV and AIDS reflects a broader commitment to more and better evaluation, as a key instrument for learning what works most effectively for poor people. The real proof of sustained value of this evaluation is in the longer-term translation of evidence into policy and practice, and through shaping future evaluation and research priorities. In particular more evidence needs to be generated on the complex pathways to achieving health impacts, and more systematic effort is required to quantify the benefits and costs of interventions.

The UK Department for International Development (DFID) has funded communities involved in the HIV response for many years. This has not been a donor-driven imperative, but a reflection of the growing role played by community-based organisations (CBOs) and civil society in the HIV response. This role has evolved organically as the impacts and urgency of the HIV emergency were felt by people in affected communities. The critical importance of this response, the ownership by and empowerment of communities that has emerged are to be celebrated.

But there is still much we do not know about the complex inter-relationship between community engagement and impact on HIV outcomes. Over the years numerous assumptions have been made about the pathways between community action and results, but apart from the existence of anecdotal evidence, these remain largely untested. This is in part a reflection of funding constraints: many community-based interventions receive limited funding and this has inevitably restricted funding for related research and evaluation.

In an era of flatlined HIV funding, where smaller CBOs and civil society are struggling to find resources, we need a much clearer understanding of how best to support and sustain effective and efficient responses which have significant impacts on affected individuals and communities. The DFID-funded World Bank evaluation that is the subject of this special issue of AIDS Care is an attempt to start building credible evidence in answer to these questions. We need to know what results our investments have produced at the community level, and how best to contribute in the future to more efficient and effective responses, while maintaining a core focus on equity.

In recent years DFID's approach to evaluation has been completely transformed. It has committed to more and better evaluations as a key instrument for learning what works most effectively for poor people. Evaluation is also a critical accountability mechanism both for the taxpayer and the organisations who receive funds alongside the people they serve. In our view, to make a difference, evaluations must deliver credible and impartial findings and recommendations, which feed directly into policies and programmes. In light of DFID's long-standing support of the community response and the lack of credible evidence in this area, we were clear that the same rigorous evaluation approach also needed to be applied in this area.

The evaluation of highly participatory and complex interventions, obviously presented significant methodological challenges. DFID considers that this evaluation is particularly interesting for the use of an innovative and bold mix of qualitative and quantitative methods to capture and interpret evidence from a range of perspectives, and to establish statistically and programmatically reliable results. This included use of experimental and quasi-experimental approaches where appropriate.

It is also notable for the highly inclusive approach to partnership working by the Word Bank/DFID evaluation team. Particularly important to the success of the evaluation was the decision at the outset to adopt a central ethos to seek beneficiary views in deciding what should be evaluated and consulting on what questions should be asked. The UK Consortium on AIDS and International Development was a strategic civil society partner in the evaluation. The Consortium facilitated significant engagement by CSOs at global, regional and national levels in the planning, implementation and dissemination of the evaluation.

The articles in this special issue provide much food for thought and critical reflection by both programmers and policy-makers. Some of the rich findings featured demonstrate the many valuable insights this evaluation has produced. But it is important to provide some pointers on how to interpret the findings and to state clearly what this evaluation is not.

The evaluation does not provide a definitive or one-size-fits-all answer to the question of whether communities contribute to changes in behaviours, knowledge, social transformation and utilisation of services. In the evaluation synthesis and the individual studies we have an enormously rich portfolio of documented information from a diverse collection of communities and geographical settings. In particular, the context-specific nature of the studies limits the direct transferability of results and presents a challenge in communicating the findings in a concise, relevant and nuanced way. In our view overstating or simplistic generalisation of the findings would be irresponsible. However, the evaluation synthesis has increased the weight of evidence towards identifying what works in regard to the community response and indicates areas for further investigation and research. It includes grading of the strength of evidence and looks at cross country comparisons.

We would like to highlight some of the important issues that warrant further attention:

Temporal changes in the dynamics of the epidemic and the contexts in which communities operate need to be better reflected in the evolving objectives of the community response and the flexibility of funding. For example, in Zimbabwe, participation in community groups was more effective in the earlier stages of the epidemic (1998–2003) than the latter stages of a more widespread epidemic. From the Kenya and Nigeria studies it could be inferred that targeted messaging may be more effective now that knowledge about HIV and AIDS has become much more prevalent following on from the broad information and communication activities that were used in earlier stages of the epidemic. It is the collective responsibility of donors, governments and communities to be responsive and adapt to the changing nature of local epidemics to shore up gains made and continue progress.

The evaluation highlights the critical importance of understanding and working with gender differences. Evidence-based targeting of interventions sensitive to gender and other vulnerabilities is important in both generalised and concentrated epidemics. In Zimbabwe group membership benefited women more than men. However, men benefited more from ART treatment in South Africa and peer mentoring for HTC sensitisation in Senegal. In Burkina Faso, participation in village committees to fight HIV/AIDS affected men's and women's knowledge of HIV differently.

The evaluation findings challenge assumptions that the community response always has positive impacts. Unintended negative impacts on stigmatising HIV-positive people were found in Burkina Faso among men exposed to prevention programmes and in Kenya as a result of home-based counselling and testing. Embedding monitoring systems that can pick up adverse effects, particularly in relation to stigma, is a necessary feature of programme design. Not surprisingly, evidence on impact on biological outcomes was mixed. Community group membership of FSW in India was associated with lower prevalence of STIs and in Zimbabwe group membership was associated with lower HIV incidence in women during the earlier stage of the epidemic. However, in Burkina Faso, Kenya and Nigeria biological outcomes were weak.

The evaluation highlights the need to have more realistic expectations of what the community response can deliver and to recognise that there may be limitations to what can be delivered. There is evidence that the community response impacted on some elements of the theory of change, but in isolation it could not achieve all the desired outcomes. Several studies revealed the importance of supportive national policies – protective policies in Kenya and Nigeria were perceived to be associated with declines in gender violence. The community response cannot substitute for weak national responses, but has a critical role to hold to account and compliment broader efforts delivered by government, private sector providers, NGOs and donors. More work is needed to tease out the roles of communities. Linking the community response more explicitly with the national response might be a way to improve effectiveness, focus and comparative advantage.

The real proof of sustained value of this evaluation is in the longer-term translation of evidence into policy and practice. It is still early days, but evaluation findings have already been used in international debates; including recent consideration by the Global Fund of community systems strengthening as a critical enabler for delivering core HIV interventions in the Strategic Investment Framework (Schwartländer et al., 2011). Various national governments have drawn on the evidence to inform discussions around their national strategic plans to address HIV. The results provide a baseline that can be used to monitor the impact of the community response in future.

This evaluation should not be seen as a one-off. In DFID's view the development community needs to commission more regular and systematic evaluations as part of a continuous process of building the knowledge base and serving as an ongoing feedback loop for programme learning. We hope that this evaluation will inform future practice in several ways. The original theory of change underpinning this evaluation is a simplified description of what is a much more complex process. More evidence needs to be generated on the complex pathways to achieving health impacts, including identifying areas where communities can be most effective, and more systematic effort is required to quantify the benefits and costs of interventions. In particular the classification of the strength of the evidence is a useful tool and could be applied to future evaluation and research that contribute to a growing body of evidence.
